# Health-seeking in fragile and conflict-affected settings: how armed violent conflict shapes maternal and child health-seeking behaviours

**DOI:** 10.1186/s12889-026-27385-2

**Published:** 2026-04-17

**Authors:** Gbadebo Collins Adeyanju, Liliana Abreu, Pia Schrage, Johanna Brinkel, Rabiu I. Jalo, Musa Muhammad Bello, Aminatu A. Kwaku, Aisha Aliyu Abulfathi, Muhammad Ibrahim Jalo, Ahmad Mahmud, Max Schaub

**Affiliations:** 1 Media and Communication Science, University of Erfurt, Erfurt, Germany; 2 Centre for Empirical Research in Economics and Behavioral Science (CEREB), University of Erfurt, Erfurt, Germany; 3 Psychology and Infectious Diseases Lab (PIDI), University of Erfurt, Erfurt, Germany; 4 Adjunct Professor of Global Health, RHIBMS University, Buea, Cameroun; 5 Department of Politics and Public Administration, University of Konstanz, Konstanz, Germany; 6 Willy Brandt School of Public Policy, University of Erfurt, Erfurt, Germany; 7 Department of Infectious Disease Epidemiology, Bernhard Nocht Institute for Tropical Medicine (BNITM), Hamburg, Germany; 8 Department of Community Medicine, Faculty of Clinical Sciences, Bayero University Kano, Kano, Kano State Nigeria; 9 Department of Community Medicine, Aminu Kano Teaching Hospital, Kano, Kano State Nigeria; 10 University of Maiduguri Teaching Hospital and University of Maiduguri, Maiduguri, Borno State Nigeria; 11 Women and Children Hospital, Damaturu, Yobe State Nigeria; 12 Modibbo Adama University Teaching Hospital, Modibbo Adama University, Yola, Adamawa State Nigeria; 13 Faculty of Business, Economics and Social Sciences, University of Hamburg & WZB Berlin Social Science Center, Berlin, Germany

**Keywords:** Maternal Health, Child Health, Health Services, Accessibility, Violent Conflict, Trust, Trauma, Displaced, Health-seeking Behaviour, Qualitative Research, Nigeria

## Abstract

**Background:**

Maternal and child mortality rates remain unacceptably high in fragile and conflict-affected settings, where insecurity, displacement, and systemic disruption hinder access to healthcare. Nigeria is one of the countries most severely affected, and continues to experience widespread maternal and neonatal deaths despite global and national efforts to achieve SDG 3.1. This study explored stakeholders’ perspectives on how armed conflicts shapes maternal and child health-seeking behaviours and outcomes in northeastern Nigeria.

**Methods:**

A qualitative approach was employed, involving Key Informant Interviews with healthcare workers, community leaders, local officials, and caregivers in three fragile and conflict-affected states: Adamawa, Borno and Yobe. These semi-structured interviews explored access to and quality of care, barriers to seeking healthcare, vaccination practices and community trust. The audio recordings were transcribed and translated, then analysed thematically in accordance with Braun and Clarke´s approach.

**Results:**

The analysis identified five interrelated themes on how armed conflict shaped maternal and child healthcare seeking behavior. Prolonged insecurity generated psychological trauma, stress-related illnesses, malnutrition, and infectious disease outbreaks, particularly in Internally Displaced Camps. Health systems collapsed due to facility destruction, workforce flight, and drug shortages, limiting service availability. Access was further constrained by poverty, insecurity, transport barriers, gender norms, low literacy, and negative provider attitudes. These challenges contributed to unsafe deliveries, increased maternal and child mortality, declining immunisation coverage, and a heightened fear of vaccine side effects amid misinformation. While trust in frontline health workers often persisted, confidence in government institutions declined.

**Conclusion:**

Armed violent conflict undermines maternal and child health through compounded psychosocial, structural, and behavioural pathways. To restore equitable service use in conflict-affected settings, it is essential to leverage residual trust in local healthcare workers, alongside community-based, conflict-sensitive strategies that address issues such as fear, costs, gender norms, transport and vaccine misinformation.

**Supplementary Information:**

The online version contains supplementary material available at 10.1186/s12889-026-27385-2.

## Introduction

Globally, maternal and child health has improved over the past two decades, yet this progress is insufficient and uneven [1]. Between 2000 and 2020, the global maternal mortality ratio (MMR) fell from 339 to 223 per 100,000 live births, however, this pace is far below the annual reduction of 6.4% required to meet the target of Sustainable Development Goal (SDG) 3.1 (< 70 per 100,000 by 2030) [1, 2]. About 800 women still die each day from largely preventable pregnancy-and childbirth-related causes, predominantly in low- and middle-income countries (LMICs), where antenatal care (ANC), skilled birth attendance (SBA) and postnatal care coverage remain inadequate [3–5]. Although child survival has improved, preventable mortality persists, with an estimated 4.8 million under-five deaths and 1.9 million stillbirths in 2023 [6–8]. The most common causes include haemorrhage, malaria, and prematurity – conditions that are largely preventable with equitable access to quality care [6, 9, 10].

These inequities are concentrated in Fragile and Conflict-Affected Settings (FCAS). Over 930 million people live amid armed conflict, displacement or instability, which damages infrastructure, depletes supplies, disrupts workforce availability and restricts mobility, all of which depress attendance at ANC, skilled delivery, postnatal care and immunisation [2]. On average, MMRs in FCAS reach 583 deaths per 100,000 live births, which far exceeds the global averages [2]. Evidence from recent conflicts shows a steep decline in ANC utilisation, alongside an increase in unattended births and maternal and neonatal complications [8, 11]. In Ethiopia, for example, conflict decreased ANC utilisation by 70% and more than halved the rate of SBA [8, 11]. Similarly, prolonged violence and gender-based insecurity in the Democratic Republic of Congo (DRC) contribute to one of the world´s highest MMRs at 473 per 100,000 live births, compared to 241 in Angola and 248 in Rwanda [5]. Disruptions to routine immunisation also contribute to outbreaks of vaccine-preventable diseases (VPDs). Global DTP3 coverage has stagnated at around 84%, with approximately 40% of under- or unvaccinated children living in conflict-affected countries [12]. Between 2010 and 2015, sixteen conflict-affected countries, including Afghanistan, the DRC, Ethiopia, Nigeria, Somalia, South Sudan and Yemen, accounted for 67% of global polio cases and 39% of global measles cases [13]. These patterns illustrate how insecurity perpetuates service disruption and reverses progress towards global health targets.

Nigeria exemplifies these dynamics. Despite gains in the national DTP3 vaccine coverage, rising from an estimated 26% in 2000 to about 60% in 2023, significant disparities remain at the subnational level, particularly across conflict-affected northern states, where insecurity, poverty, distance, and costs impede access to services [14]. The Boko Haram insurgency has damaged health facilities, displaced healthcare workers, and undermined outreach and vaccination services, leading to widespread reliance on Traditional Birth Attendants (TBAs) and informal providers, athough equally important [15]. Consequently, maternal and neonatal mortality remain among the world´s highest (MMR > 800 per 100,000 live births and neonatal mortality at 33 per 1,000), and trust in health institutions remains fragile [9]. About 70% of maternal deaths occur in sub-Saharan Africa (SSA), and nearly 80% of stillbirths occur in SSA and Southern Asia, where women are six to eight times more likely to experience a stillbirth than in Europe or North America [6, 7, 16]. If current trends continue, over one million additional women risk losing their lives by 2030 [7]. Overall, investing in maternal and newborn health is not only lifesaving, it is also economically sound at all levels — global, regional and national — because for every US$1 invested, between US$9 and US$20 can be returned [10].

While quantitative data delineate the scale of the problem, the perspectives of those navigating healthcare services in FCAS are underrepresented. The study hypothesised that stakeholder narratives have tendency to clarify how insecurity, gender and cultural norms, financial constraints, transport barriers, healthcare worker attitides, and misinformation interact to shape behaviours and outcomes, as well as how trust is built or eroded. This study therefore explores stakeholders’ perspectives on maternal and child health-seeking behaviours and outcomes in conflict-affected communities in northeastern Nigeria. By centring on lived experience, the study aims to inform context-sensitive, resilient strategies to strengthen ANC, skilled delivery, postnatal care, and immunisation in FCAS.

## Methods

### Study design and setting

**Fig. 1:**
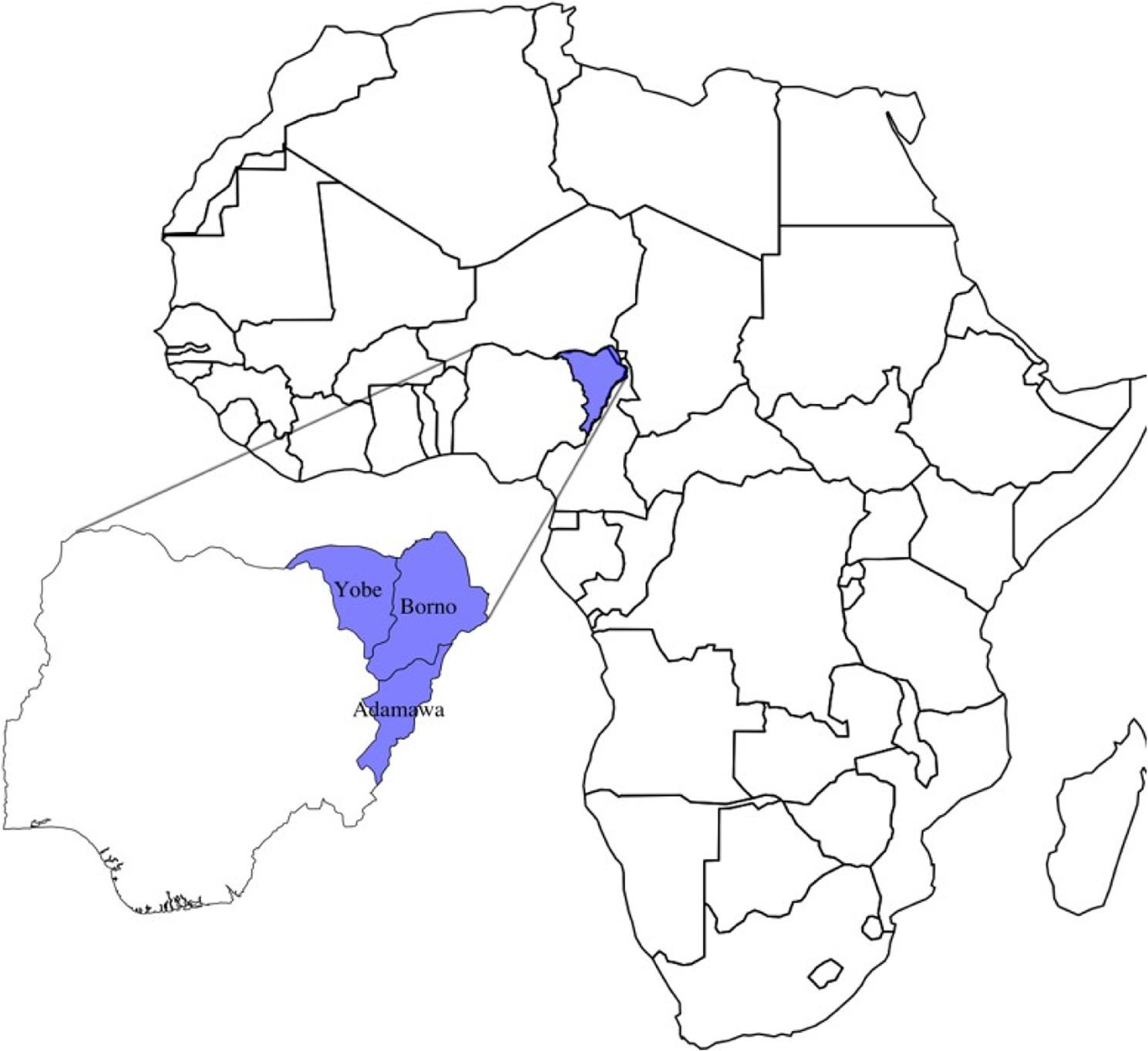
Overview of study settings

This study employed a qualitative design involving Key Informant Interviews (KIIs) to explore maternal and child health-seeking behavious and associated outcomes in conflict-affected communities in northeastern Nigeria. The study was conducted across three states in the northeast of Nigeria, as shown in Fig. 1: Adamawa, Borno and Yobe - which have been severely impacted by the Boko Haram insurgency and associated armed violence. The varying levels of insecurity and disruption to health systems in these states provide diverse insights into the challenges of accessing and delivering maternal and child healthcare in fragile contexts.

### Study population and sampling

Participants were purposively selected to capture a broad range of experiences and roles within maternal and child health. This included healthcare workers (such as nurses, midwives, and primary healthcare officers); community and religious leaders; local government health officials; caregivers; and mothers of young children. Participants were eligible if they had lived or worked in the study area for at least one year during the ongoing conflict, and if they could speak English or Hausa. Purposive and snowball sampling strategies were employed to identify individuals with first-hand knowledge of maternal and child health issues in the conflict-affected areas.

Participants were approached through trusted community gatekeepers, including local leaders and healthcare workers, to ensure safe and culturally appropriate engagement in a high-conflict setting. Recruitment was conducted cautiously and flexibly, with interviews scheduled only when security conditions permitted and in locations agreed upon as safe and accessible by participants.

### Data collection

A total of 30 KIIs were conducted between June 4–29, 2024 with key stakeholders in the northeast of Nigeria, the region most affected by the Boko Haram insurgency, particularly in the states of Adamawa, Borno and Yobe. Semi-structured interview guides (see supplementary file) were used to focus on participants´experiences, expert opinions, and perceptions of the impact of conflict on: (1) Access to and quality of maternal and child healthcare services; (2) Barriers and facilitators to healthcare-seeking behaviour; (3) Vaccination coverage and community attitudes towards immunisation; and (4) Trust and relationships between communities and healthcare providers. The interviews were conducted face-to-face in private, and safe locations - typically healthcare facilities in the study area - depending on accessibility and security considerations. The interviews were conducted in local languages, with participants’ consent, and audio-recorded for transcription, first in Hausa and then in English. They were also supplemented with detailed field notes. Each interview lasted approximately 60 min and was conducted by trained researchers familiar with the local context. Interviews with women were conducted by female researchers and interviews with men by male researchers.

### Data management and analysis

The interviews were transcribed verbatim from Hausa into English by experienced researchers. The KIIs were analysed using hybrid coding, combining deductive and inductive content analysis in MAXQDA software [17, 18]. The transcripts were verified for accuracy by bilingual researchers. Content analysis followed the steps outlined by Clarke and Braun [19]. While most coding and theme development adhered to the deductive, some codes and themes were generated inductively. The codes/themes were selected a priori based on the study objectives. Participants were anonymised and coded based on their state of residency (e.g., Borno), local government area (e.g. Damaturu, Jada, etc.) and a number identifier (e.g., 1, 2, 3, etc.). Participants were identified for example as Borno_Magumeri3, Yobe_Potiskum3, Adamawa_Madagali3, and so on.

### Ethical considerations and reporting standards

Ethical approval was obtained from the Nigerian National Health Research Ethics Committee (NHREC). Written informed consent was obtained from all participants prior to the commencement of the data collection. Participants’ data were anonymised for confidentiality purposes, and they were informed of their right to withdraw at any time without consequence or explanation [20].

### Reflexivity statement

This qualitative study was conducted in a conflict-affected setting among vulnerable populations, necessitating heightened reflexivity and ethical consideration. Interviewers were selected based on their academic qualifications and experience working in humanitarian contexts. They received training in qualitative interviewing, trauma-informed approaches, and non-leading interviewing techniques. Interviewers were fluent in local languages and culturally competent, facilitating trust and rapport. Strict safety and ethical protocols were applied throughout the study, including context-specific risk assessments. Regular debriefings and supervisory support ensured ongoing reflexivity, researcher well-being, and adherence to ethical standards during data collection and analysis.

## Results

The analysis of data revealed five major themes illustrating the influence of armed conflict on behaviours and associated outcomes realating to maternal and child health-seeking: The health and psychosocial impacts of conflict; the collapse of healthcare services and infrastructure; barriers to healthcare access and utilisation; the effects on maternal and child health and immunisation; and trust, attitudes and relationships within the healthcare system.

### Health and psychosocial impacts of armed conflict

Participants described how prolonged insecurity and violence had led to psychological trauma and stress-related illnesses, as well as worsening poverty and malnutrition. Feelings of fear, anxiety, and hopelessness were widespread and contributed to mental and physical health challenges.

*“Some of the concerns I have observed in this community due to the insurgency were anxiety*,* fear*,* gunshots*,* and rampant abductions. Also*,* we fear that at any moment we could die*,* at times you will see food*,* but could not eat because of fear of being attacked by Boko Haram members” (KII_Borno_MMC4). “The most common health concern I have observed in this community during the violence*,* is that people have been psychologically terrorised” (KII_Borno_Magumeri4). “Some of the common health concerns I have observed in this community due to the insurgency are psychological problems caused by gunshots and bombs*,* as well as depression experienced by members of the community” (KII_Borno_MMC2)*.

Respondents also reported an increase in non-communicable diseases, particilarly hypertension, which they attributed to chronic stress and insecurity.

*“Almost every adult in this village has hypertension” (KI_Adawama_Madagali4). “It’s as a result of this insurgency that I developed grey hair and high blood pressure. There are several people that have experienced similar health issues during the insurgency” (KII_Yobe_Potiskum5). “Some people were also affected by high blood pressure and related health issues” (KII_Yobe_Damaturu2).*

Malnutrition emerged as another major health effect. The destruction of livelihoods and restricted mobility led to hunger and undernutrition among pregnant women and children.

*“First*,* there is the issue of poverty and hunger*,* which also results in malnutrition in children” (KII_Adawama_Madagali1). “We are faced with difficulties accessing care due to the insecurity situation in the community*,* and we also don’t have enough food to eat.” (KII_Borno_MMC5).*

Participants also described frequent outbreaks of infectious diseases such as malaria, cholera, measles and diphtheria in internally displaced persons (IDP) camps, attributing these outbreaks to overcrowding, poor sanitation and the breakdown of preventive services.

*“The most common health issue I observed in this community was the cholera outbreak because of open defecation*,* and many lives were lost due to such outbreaks” (KII_Borno_Magumeri3). “The health issues we faced were malaria*,* fever*,* tuberculosis*,* whooping cough*,* measles and meningitis…these were our main problems” (KII_Borno_MMC5). “Presently*,* there is an outbreak of diphtheria and measles*,* which is due to inability of community members to access healthcare*,* including vaccinations” (KII_Yobe_Damturu1). “Our stay in IDP camps affected our people’s health*,* because the place was crowded*,* leading to outbreaks of cholera*,* malaria and other infectious diseases*,* which predominantly affected children and women” (KII_Yobe_Damturu2).*

### Collapse of healthcare services and infrastructure

The armed conflict has severely weakened healthcare systems across the study population. Particpants reported the widespread destruction of facilities, the looting of medical supplies and the flight of healthcare workers and personnel.

*“The population of Potiskum is too large for the number of functioning hospitals…at times we used to take our patients to Azare*,* Nguru*,* and Gombe due to the scarcity of healthcare and healthcare workers” (KII_Yobe_ Potsikum3). “Another reason that has changed health-seeking behaviour is the lack of drugs…even if you come to access care*,* there is no medicine” (KII_Adawama_Madagali1). “Exposure to violence has affected people’s seeking behaviour in this community because*,* at that time*,* there were no qualified doctors to attend to our patients - everyone had run away. Our healthcare facilities were shut down*,* so we were unable to access healthcare” (KII_Borno_MMC5).*

Healthcare facilities were frequent targets of insurgent attacks by Boko Haram, creating an environment of fear among patients and healthcare workers.

*“When a pregnant woman was due to give birth*,* we would use the services of TBA to assist with the delivery. Unfortunately*,* we could not get any healthcare worker to assist in fixing IV fluids/drip for a sick person. And whenever they came*,* they would loot all the drugs in the hospital and leave” (KII_Borno_Magumerii5). “During that time*,* access to healthcare services became very difficult because the hospitals have been burnt down or no longer functional and there are no means of transport” (KII_Adawama_Madagali5). “We had about 25 healthcare facilities in our community*,* but due to the Boko Haram insurgency*,* all of those hospitals were closed down” (KII_Borno_Mogumeri1).*

These disruptions left many communities without functioning healthcare facilities, contributing to multiple barriers to healthcare access and utilisation.

### Barriers to healthcare access and utilization

Participants described the complex interplay of social, economic, and structural barriers limiting access to care during the conflict. Fear of attacks and insecurity were the most frequently cited constraints.

*“Some people who live far away could not come and access healthcare for fear of being attacked or kidnapped by Boko Haram…some*,* the distance from their area to the hospital is about 50km” (KII_Borno_Magumeri1). “Another thing that affected the health issues was that people could not come out to access services due to fear of an attack on the way to hospital” (KII_Yobe_Potiskum2).*

The majority of people in the region have lost their homes and livelihoods due to the insurgency. The resulting poverty diminishes their self-agency to access healthcare services. Much of the population in the study areas lacks sufficient resources to meet their basic needs, which affects how healthcare is sought.

*“One of the barriers that prevent an individual from seeking healthcare services was poverty” (KII_Yobe_Potiskum5). “Poverty prevents them from pre- and postnatal appointments…e.g.*,* she wakes up without anything to eat and with no means of transport to the hospital for follow-up appointments. This prevents them from following-up on their postnatal appointments.” (KII_Borno_Magumeri4). “One of the major barriers that prevent people from seeking care was poverty of survival.” (KII_Adamawa_Jada5).*

Even when services were available, the cost of care deterred people from seeking it.

*“When sick*,* people could not go to the hospital because they had no money to pay*,* and this was as a result of the insurgency. Yes*,* health-seeking behaviour has changed due to the lack of money to pay for medical expenses.” (KII_Yobe_Potiskum3). “Another barrier is financial constraints*,* where people cannot pay for medical services even if they go to the hospital” (KII_Borno_Magumeri1). “…these days*,* the cost of drugs is just too high for ordinary people” (KII_Adamawa_Jada1).*

Gender and sociocultural norms also limit healthcare uptake. In most households in the study settings, the male head of the household makes the healthcare decisions, including those related to maternal and child healthcare. Therefore, a husband’s attitude (approval or disapproval), which is usually influenced by cultural and religious biases, significantly affects access to maternal and child healthcare in these settings.

*“We discovered that the majority of husbands do not allow their wives to take their children for vaccination due to traditional beliefs*,* ignorance*,* and illiteracy on their part” (KII_Borno_Magumeri3). “Despite free drugs and free delivery services being provided for pregnant women*,* the only other issue we are experiencing is that*,* during antenatal care (ANC) appointments*,* husbands do not allow their wives to attend” (KII_Yobe_Damaturu4).*

Low literacy and limited awareness of the health benefits further discouraged people from care-seeking.

*“One of the main barriers preventing individuals from accessing healthcare in this community is ignorance or illiteracy on the part of the people” (KII_Yobe_Potiskum4). “What usually prevents them from attending antenatal and postnatal appointments is that*,* when they feel physically strong after childbirth*,* they think they are OK and decide not to attend postnatal appointments*,* due to ignorance and illiteracy” (KII_Borno_Magumeri3). “The common health challenge in this community is a lack of awareness of immunisation services” (Borno_Magumeri4).*

Participants also reported negative treatment and unprofessional behaviour, and a lack of discipline among healthcare workers in the study settings. This ranged from bad attitudes towards mothers to feeling judged by healthcare workers for their health conditions, among other things. Such negative experiences discourages patients from seeking healthcare at these facilities in the future.

*“There is a need to improve the attitude of healthcare workers on the way they treat patients. The ways and manner they shout at patients is very bad and uncalled for” (KII_Borno_Magumeri2). “One of the barriers that prevent people from seeking healthcare services is bad attitude of healthcare workers” (KII_Borno_MMC3). “The attitude of healthcare workers is one of the barriers that prevent individuals from accessing healthcare in this community. Also*,* the delay in attending to patients when they visit the hospital” (KII_Yobe_Damaturu1).*

Incessant unavailability, poor perception and unreliability of conventional healthcare facilities heightens people’s beliefs in local traditional herbal approaches for managing health.

*“Some prefer to visit a traditional herbalist for care” (KII_Adamawa_Jada4) “There are those who do not believe in the Western medication*,* they only believe in traditional and herbal medications” (KII_Yobe_Potiskum2).*

Finally, transportation challenges were a recurrent obstacle, especially for rural populations.

*“Some do not have the means to transport their children for immunisation because everything they had was looted by Boko Haram and they don’t have a health facility close to them.” (KII_Borno_Magumeri5). “…others include lack of effective transport*,* food*,* etc.” (KII_Adamawa_Jada3). “The lack of mobility to transport a pregnant woman in labour to hospital is one of the barriers that prevents them from seeking healthcare there” (KII_Yobe_Damaturu5).*

### Maternal and child health outcomes and immunization

Respondents consistently reported a deterioration in maternal and child health outcomes. The conflict not only made childbirth dangerous, but also impossible. As well as having limited access to healthcare facilities, SBAs are a prime targets for insurgent attacks. The demand for TBAs increases during the times of active armed conflict. In addition, the armed conflict makes night-time travel unsafe, making it nearly impossible for some communities to get help when giving birth at night.

*“Due to the conflict*,* in this community*,* a woman went into labour at night*,* with no transportation to the hospital for delivery*,* the labour turned out to be very difficult*,* with a lot of bleeding. While the woman survived*,* we lost the baby” (KII_Adamawa_Jada1). “I observed in communities affected by the conflict*,* you can see pregnant women delivering babies on the road or anywhere because there are too many security roadblocks in town. As a result*,* many women and children have been lost” (KII_Adawama_Jada4)*.

High child mortality and malnutrition were both linked to the same barriers.

*“Yes*,* the barriers I mentioned contributed to the high mortality rates among children. In my own unit at the malnutrition centre*,* I witnessed so many children dying because of these barriers*,* including a lack of routine immunisation” (KII_Adawama_Jada5). “…the cause of neonatal mortality is a lack of courage among caregivers…when their children are sick*,* they don’t want to take them to hospital” (KII_Adawama_Madagali5).*

Vaccination coverage has declined due to insecurity, misinformation and religious or traditional beliefs.

*“Vaccination coverage in this community has declined because the healthcare workers are not coming as often as they did in the past” (KII_Yobe_Damaturu3). “Vaccination coverage has been affected because*,* unlike in the past*,* healthcare workers no longer visit house-to-house to vaccinate children” (KII_Yobe_Damaturu2). “People in this community who do not allow their children to be vaccinated do so out of ignorance and deep-rooted cultural beliefs” (KII_Adawama_Madagali2). “There is a high dropout rate for vaccinations in this community…they used to skip some days and then stop completely due to a lack of knowledge and misinfrmation. So*,* we are experiencing a lot of defaults on this side” (KII_Yobe_Damaturu1).*

The role of the fear of adverse events following immunization (AEFI) was found to be an important driver of health-seeking behaviour for childhood immunisation in the study settings. The trauma or exposure to conflict may have exacerbated this fear of AEFI.

*“In the past*,* since the conflict*,* some people in this community did not allow their children to be vaccinated*,* because of the fear of side effects” (KII_Adamawa_Jada1). “While those who do not allow their children to be vaccinated do so because of fear of side effects*,* since the start of the conflict” (KII_Borno_MMC1). “Some also do not want their children to be vaccinated due to the adverse effects of the vaccines” (KII_Adamawa_Jada4).*

The study observed the entrenched misinformation that was amplified by the armed conflict situation. Vaccination became a propaganda tool used by both the insurgent group and the Nigerian military. The insurgent group accused immunisation vaccinators of being spies for the Nigerian government and, in some cases, kidnapped or killed them. A common conspiracy theory about routine immunisation was that it was a form of family planning therapy intended to render children infertile.

*“One of the reasons is the efficacy of the vaccine and rumours that it is being used to depopulate people in the community” (KII_Borno_Magumeri5). “Some believe in the rumours that the vaccinations are being used on our children for the purpose of family planning…meaning they won’t be able to have children in the future” (KII_Adamawa_Jada2). “Some people do not allow their children to be vaccinated because they suspect that it is a plot by Westerners to depopulate the community*,* using the vaccines and that these vaccinated children will not survive beyond a certain number of years” (KII_Yobe_Potiskum4)*.

Religious belief was shown to be a factor driving hesitation about child immunisation. In addition to misinformation, religious belief was shown to drive lower demand for vaccination within the population.

*“One reason why some people decide not to vaccinate their children is religious belief”* (KII_Adamawa_Jada2). *“One of the factors preventing caregivers from taking their children for vaccination is religious belief*,* and they don’t take hospital drugs as medicine”* (KII_Yobe_Potiskum1).

Traditional beliefs were often cited as a reason for not vaccinating children. These beliefs ranged from a preference for herbal medicine over conventional Western medicine, to beliefs about the use of natural ingredients and avoidance of injecting chemicals into the body.

*“They do not want to vaccinate their children due to their traditional beliefs” (KII_Yobe_Damaturu1). “Some believe in traditional medicine and usually seek a herbalist for healthcare” (KII_Borno_MMC2). “There is a traditional belief among those who do not vaccinate their children…that*,* in their tradition*,* families do not allow their children to be vaccinated” (KII_Adamawa_Jada5).*

### Trust in and relationships with healthcare systems

Despite the ongoing insecurity, most participants continued to expressed their trust in, and appreciation of, the commitment of local healthcare workers.

*“Exposure to violence hasn’t affected our relationship with healthcare workers; we maintain a good relationship with them” (KII_Adamawa_Jada1). “There is a good relationship between healthcare workers and the communities because they are fully aware of the importance of accessing healthcare services” (KII_Borno_Magumeri4). “Our exposure to violence does not change the relationship with healthcare workers” (KII_Yobe_Potiskum3).*

In contrast, trust in government institutions has eroded due to insecurity and a lack of support. Some people believe that the government is not doing enough to keep them safe, leaving them vulnerable and the provision of basic services, among other things.

*“Trust and confidence have reduced because of the insecurities and problems that have occurred” (KII_Borno_Magumeri5). “The government is not taking care of the communities*,* and there is a level of distrust among people somehow” (KII_Yobe_Potiskum3). “Yes*,* trust has been affected because people are facing financial constraints” (KII_Adamawa_Madagali1).*

Despite the above sentiment expressed by the majority of participants, violent conflict has had a negative effect on the relationship between mothers and healthcare workers in particular. This is primarily because trust is difficult to establish or maintain in times of violent conflict.

*“Yes*,* the relationship with healthcare workers has changed*,* and this is further caused by the attitude of the healthcare workers during these times” (KII_Adamawa_Madagali1). “Yes*,* the relationship with healthcare workers has changed because we live in fear and trust is minimal…you don’t know who you are interacting with anymore” (KII_Yobe_Potiskum5). “We trusted the healthcare workers we interacted with in the past*,* but we don’t trust or have confidence in the new ones that they have introduced or deployed to our health facility because of security reasons” (KII_Borno_Magumeri5).*

## Discussion

This study demonstrates that armed conflict has an impact on maternal and child healthcare-seeking behaviors and outcomes through a cascading and mutually reinforcing set of psychosocial, structural, economic and relational disruptions, rather than through a single pathway. Participants described widespread psychological trauma, chronic stress, hypertension, poverty, and malnutrition, as well as recurrent infectious disease outbreaks in overcrowded and under-resourced settings. This illustrates how insecurity penetrates everyday health and wellbeing. Psychosocial factors (e.g. fear and trauma) constrain mobility and decision-making, while structural breakdowns in healthcare services and infrastructure limit service availability, and economic hardship exposes households to vulnerability in the event of direct and indirect systems breakdown, and these factors interact in a mutually reinforcing, cascading manner that amplifies vulnerability and progressively restricts access to maternal and child healthcare. At the systems level, the destruction of facilities, the looting of supplies, the flight of health workers and targeted attacks on infrastructure led to the collapse of service delivery, prompting people to rely on TBAs and informal providers. These structural failures, intersecting with poverty, transport barriers, gendered decision-making norms, low literacy and negative provider attitudes, further suppress care-seeking, particularly with regards to ANC, facility delivery and childhood immunisation. The findings also show how conflict amplifies misinformation, fear of adverse events, religious and traditional beliefs, and politicised rumours around vaccination. This contributes to declining coverage and high dropout rates. Importantly, while trust in local frontline health workers often persisted, trust in government institutions was eroded and relationships with facilities became more fragile. By integrating psychosocial, health system, sociocultural, and trust dimensions, this study advances existing knowledge beyond linear “conflict-damages-services” explanations, demonstrating instead how protracted insecurity produces a complex ecology of barriers that collectively drive adverse maternal and child health outcomes.

### Conflict, mental health and health-seeking

The widespread psychological distress, hypertension, and malnutrition reported by participants reflect the far-reaching consequences of armed violent conflict on health and wellbeing. Similar patterns of anxiety, depression, and stress-related non-communicable diseases have been reported among displaced populations in other conflict-affected settings, such as Ethiopia’s Tigray region and the Democratic Republic of Congo [21, 22]. Scholars have confirmed these associations globally, showing that conflict exposure is linked to higher maternal and child mortality, decreased vaccination coverage, and reduced healthcare-seeking [23–26]. Building on this body of work, our findings demonstrate how fear and trauma can manifest as behavioural avoidance. For example, pregnant women may choose not to attend ANC appointments or give birth in healthcare facilities due to feelings of insecurity and anxiety.

### Collapse of healthcare systems

Participants´accounts of facility destruction, workforce flight and medicine shortages mirror the hallmarks of service collapse in FCAS identified by other studies [27, 28]. In such environments, supply chains are interrupted, human resources are depleted, and service delivery becomes fragmented. For example, during the conflict in Tigray, Ethiopia, more than half of health facilities became non-functional and maternal health coverage fell by nearly 60% [22, 29]. The present study´s qualitative insights illustrate the lived consequences of this collapse, including long distances to operational facilities, frequent stockouts and reliance on TBAs in the absence of healthcare workers and hospital staff.

### Barriers to healthcare access and utilization

Fear, distance and poverty emerged as additional and compounding barriers to maternal and child healthcare. These findings align with evidence from SSA demonstrating how insecurity interacts with socioeconomic inequality to suppress healthcare utilisation [24, 30]. Poor health literacy and negative attitudes among healthcare workers further discouraged healthcare-seeking behaviour and reinforced the underutilisation patterns described in multi-country reviews and studies [24, 26, 27, 31–35]. Together, these barriers reveal that conflict not only reduces the supply of healthcare services, but also undermines demand through fear, knowledge-gap, and the erosion of agency.

### Maternal and child health and immunization outcomes

The deterioration in maternal and child health outcomes reported in this study is consistent with the findings of other studies showing that armed conflict increases maternal mortality, reduces ANC coverage, and lowers vaccination rates [23–26, 36]. In our context, vaccination behaviour was particularly shaped by misinformation, including rumours about infertility and Western depopulation conspiracies, which contributed to vaccine hesitancy. Similar narratives were found in other studies, where distrust and insecurity were found to drive immunisation decline across FCAS [24, 31]. These behavioural dynamics reinforce the idea that conflict affects healthcare through systemic and psychosocial pathways, disrupting supply chains while simultaneously fuelling fear and rumours.

### Trust, relationships, and the social ecology of health

A notable finding of this study is that trust between communities and frontline healthcare workers has persisted despite widespread institutional distrust of the government. This is consistent with evidence that interpersonal trust often endures in fragile contexts, even when institutional credibility collapses [30]. Such relationships can lay the foundation for community resilience and post-conflict recovery. Strenthening these relationships through community healthcare workers, participatory governance and continuity of care could help rebuild utilisation of, and confidence in, formal health systems.

### Impacts of religious beliefs

In most places in the SSA region where misinformation about vaccination is found, it is usually closely linked to religious belief [33, 34, 37–41]. Misinformation and religious beliefs can amplify vaccine hesitancy, reducing demand for vaccinations in populations with limited resources, as is the case in the northeast Nigeria. Furthermore, a stronger bond has been found between these two factors, particularly in Muslim-dominated regions such as northeast Nigeria [32, 33, 41]. The impact of religious beliefs pose a greater threat to the utilisation of maternal and child health services, particularly immunisation services, in Muslim-dominated areas such as the study setting. This outcome is not unrelated to a previous finding that showed an association between religious belief and immunisation in the Muslim-dominated northeast Nigeria, where the current study was conducted [31, 33].

### Gender, sociocultural norms and conflict-driven constraints on healthcare access

In the study settings, gender and sociocultural norms are deeply intertwined with the dynamics of armed conflict, collectively intensifying barriers to maternal and child healthcare behaviour and outcomes. In patriarchal household structures, which predominate in these settings, men retain primary decision-making authority over women’s mobility and healthcare use [33, 40, 41]. These norms become more restrictive in conditions of insecurity. Armed conflict exacerbate this control as families adopt protective strategies that restrict women’s movement due to fear of violence, abduction or harassment by armed groups, thereby reducing their ability to seek care independently. Furthermore, abduction of women by Boko Haram has been systematically deployed as a weapon of armed conflict, to instil fear, exert territorial control and reinforce gendered domination [42]. Disrupted education systems, displacement, and heightened poverty also exacerbate illiteracy and reliance on traditional and religious beliefs, further shaping male attitudes towards formal healthcare. In this context, refusal of antenatal care or childhood vaccination is a manifestation of structural vulnerability where gender norms, fear and institutional breakdown converge, rather than being solely a product of individual belief. As reflected in participants’ accounts, male disapproval, often based on misinformation, sociocultural norms, and reinforced by conflict-related uncertainty, directly constrain women’s access to essential services. This illustrates how sociocultural and conflict-related factors interact to perpetuate health inequities and increase the risks faced by women and children.

### Implications for practice and policy

The findings underscore the urgent need for context-specific, conflict-sensitive health strategies. Firstly, community-based and mobile outreach models are essential for accessing populations affected by insecurity and geographical isolation. Evidence from intervention reviews in FCAS indicates that mobile teams, local volunteers and community-based health posts can maintain continuity of care during crises [27, 28]. Secondly, psychological and livelihood support should be prioritised for integration into maternal and child health programmes. The observed link between stress, hunger, and care-seeking behaviour mirrors the findings of scoping reviews which emphasise that socio-economic and mental health interventions must accompany healthcare or hospital services in order to improve uptake [31]. Thirdly, empowering local healthcare workers who retains the community´s trust is central to post-conflict settings and rebuilding efforts. Training, adequate remuneration, and security protections can help sustain their presence in hard-to-reach areas. Fourthly, gender-inclusive programming that engages men in maternal and child health decisions could reduce barriers created by patriarchal norms. Fifth, engaging religious and community leaders, as was successful in Nigeria´s polio-eradication strategies, may improve acceptance of maternal and child health interventions. Involving religious leaders as immunisation programme anchors or ambassadors could add value to any future interventions in the area. Finally, policy frameworks for Nigeria and other FCAS should explicitly link maternal and child healthcare services with peacebuilding and governance reforms. Integrating these efforts into national healthcare plans would contribute to achieving Sustainable Development Goals 3.1 (reducing maternal mortality) and 3.2 (improving child survival), as well as Goal 16 (promoting peace and building resilient institutions).

### Methodological considerations

The study has several limitations. Firstly, the findings were derived from only three conflict-affected states and may not represent the views of all FCAS or other regions of Nigeria. Participant recruitment depended on local access and security conditions, which may have excluded more remote or severely affected areas. As with all qualitative research, the findings are context-specific and reflect the perspectives of the participants. The temporal gap between data collection and the current state of the conflict should be considered when interpreting how these findings apply to the present-day situation, giving the evolving nature of insecurity. However, this gap does not diminish the study’s value. Furthermore, methodological rigour was ensured through triangulating participant categories, achieving thematic saturation, and conducting peer debriefings among researchers. Ethical considerations were maintained throughout to preserve the authenticity of the participants´voices.

## Conclusion

This study contributes new understanding by moving beyond linear explanations of service disruption to conceptualise a dynamic, multi-layered “social ecology” through which armed conflict reshapes maternal and child health. The findings demonstrate how interconnected factors such as psychosocial distress, non-communicable disease risks, health system breakdown, sociocultural norms, misinformation and evolving trust relationships collectively suppress care-seeking and exacerbate poor outcomes by integrating them within a unified analytical framework. Importantly, the study draws attention to the simultaneous erosion of institutional trust and the persistence of interpersonal trust in frontline health workers, shedding light on under-examined pathways for resilience and system recovery. This relational and systems-oriented approach deepens existing scholarship and offers a stronger conceptual foundation for conflict-sensitive programming in maternal and child health.

**Fig. 2:**
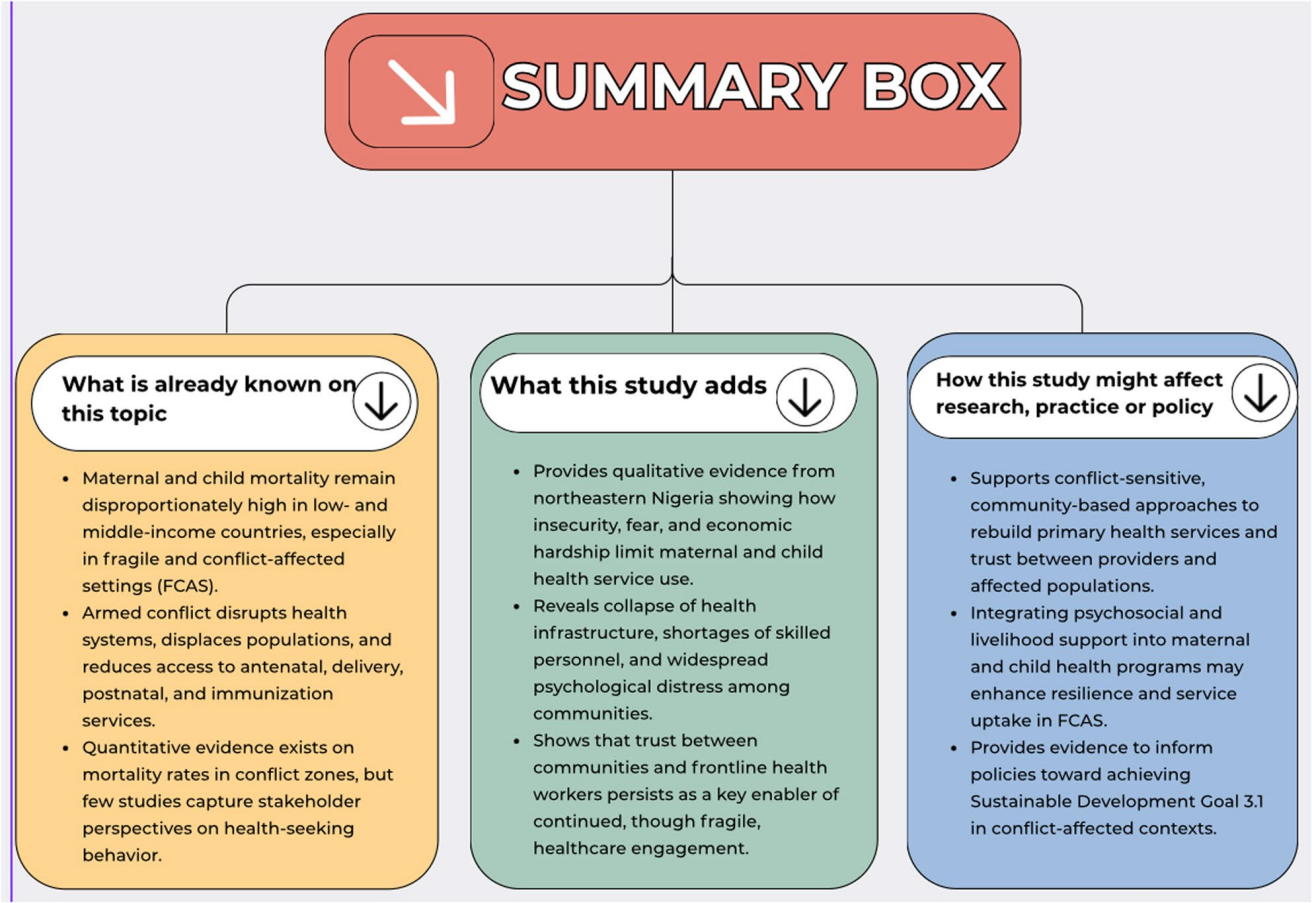
Summary box of the study

Armed violence in northeastern Nigeria continues to significantly undermine access to and utilisation of maternal and child health services. As shown in Fig. 2, this study adds rich qualitative evidence to the existing literature on maternal and child health in FCAS. Consistent with prior research, insecurity, fear, displacement, social fragmentation and economic hardship emerged as major constraints to accessing and utilising healthcare. The findings further document the partial collapse of health infrastructure, critical shortages of skilled personnel, and widespread psychological distress within communities. Together, these conditions weaken health-seeking behaviours and hinder the effective delivery of healthcare services.

While earlier studies have extensively documented the decline in service coverage, this study advances the field by unpacking the behavioural, psychosocial and structural mechanisms that perpetuate reduced care-seeking, even in areas where some services have been restored. These mechanisms include trauma, persistent fear, misinformation, gendered decision-making norms and institutional distrust. Notably, the study identifies a dynamic that has been under-explored in the literature on conflict and health: the endurance of interpersonal trust between communities and healthcare workers despite systemic collapse. Although distrust of government institutions was widespread, trust in local healthcare providers often remained intact, enabling continued engagement with services. These findings underscore the importance of community-driven, conflict-sensitive strategies that rebuild primary healthcare systems while providing psychosocial and livelihood support. Such approaches provide a pathway towards more resilient and equitable healthcare systems in fragile contexts. Future interventions must be locally grounded, responsive to evolving conflict dynamics, and explicitly trust-based to restore progress towards Nigeria’s maternal and child health targets.

By combining behavioural insights with systemic analysis, this study bridges public health, behavioural science, and conflict studies, generating context-specific recommendations for community-based and conflict-responsive interventions. It is one of the few studies to explicitly connect health-seeking behaviour, mental health, and trust dynamics within the broader ecosystem of fragility, conflict, and resilience in Nigeria.

## Supplementary Information

Supplementary Material 1.

## Data Availability

The datasets used and/or analyzed during the study are available at [https://osf.io/3ujwz/overview](https:/osf.io/3ujwz/overview).
