# Supplementary material for: Health-seeking in fragile and conflict-affected settings: how armed violent conflict shapes maternal and child health-seeking behaviours
Source: BMC Public Health. 2026 Apr 17;26:1274. doi: 10.1186/s12889-026-27385-2 (PMC13091252; doi:10.1186/s12889-026-27385-2)
Supplement: Supplementary file 1 — Supplementary Material 1. [file 12889_2026_27385_MOESM1_ESM.pdf]

KII interview guide

Procedures:

Consent Process:

- *Explain the nature of the study.*
- *Read and explain informed consent to the participant.*
- *Assure confidentiality and anonymity.*
- *Introduce yourself and the purpose of the interview.*
- *Build rapport by expressing gratitude for their participation and explaining the importance of their insights.*

➢ Starts Audio-recording (always refer the participants if you can start recording)

Section 1. Background Information

Sub-theme 1.1 – Introduction

1. *Ask open-ended questions to help participants ease into the conversation, such as:*
   - Can you tell me a bit about your experiences and background in this community?
   - What are some of the main health challenges you've observed in this area?

Section 2. Community Exposure to Violence

Sub-theme 2.1 – Understanding the Impact of Violent Armed Conflict

2. *Explore the stakeholders' perceptions and experiences of violent armed conflict in the region:*
   - Has this community recently been affected by violence (violent events that involved armed groups), and, if so, what happened?
   - What are some common health issues or concerns you've observed in communities affected by violent conflict?

Section 3. Health Behaviors and Access to Care

Sub-theme 3.1. – Health Seeking Behavior

3. *Investigate how exposure to violence influences health-seeking behaviors:*
   - In what specific ways do you think exposure to violence affects people's health-seeking behaviors?
   - Specifically, how is maternal and child health impacted among individuals affected by conflict?
   - Could you describe how people in this community typically seek healthcare services?
   - Have you noticed any changes in health-seeking behaviors among community members following incidents of violence?
   - What factors do you believe influence whether or not individuals seek healthcare in the context of violent conflict?
   - Does exposure to violence change their relationship with healthcare providers?
   - What factors do you think influence the decision to vaccinate or not vaccinate children in your community? *Follow-up:* how about among vaccine-hesitant groups and who are they?

- Could you describe any changes in trust or confidence in the health system and the government responsible for managing it following the recent conflict? If trust has been affected, what factors contributed to this change? If not, what factors have helped maintain trust despite the conflict?

Sub-theme 3.1. – Barriers to Healthcare Access

4. *Explore the challenges or obstacles people face in accessing healthcare services due to conflict-related factors:*
    - What are some of the main barriers preventing individuals from accessing healthcare in this community?" *Follow-up:* pre and postnatal appointments.
    - How do you think these barriers contribute to the high mortality rates among children?
    - Are there any specific groups or demographics that face greater challenges in accessing healthcare?
    - Have vaccination rates been affected?

Sub-theme 3.2. – Community Resilience and Coping Strategies

5. *Investigate resilience factors and coping strategies employed by the community to mitigate the impact of conflict on health:*
    - Can you share any examples of resilience or coping mechanisms you've observed within the community?
    - How do community members support each other during times of conflict-related health crises?
    - Are there any local initiatives or programs aimed at improving healthcare access or addressing the health impacts of violence?

Section 4. Potential solutions and recommendations

6. *Gather stakeholders' perspectives on potential interventions or strategies to address the health challenges exacerbated by conflict:*
    - What do you think could be done to improve healthcare access and reduce child mortality rates in this context?
    - Are there any specific resources or support systems that you believe would be beneficial for the community?
    - Are there any security-related measures that could be taken to improve access to healthcare for mothers and children?
    - How can stakeholders at various levels collaborate to address the health needs of conflict-affected populations?

Closing Remarks:

Thank the participant for the valuable insights and contributions.

Offer an opportunity to add any final comments or reflections.

Inform of the next steps in the research process and express your willingness to follow up if needed.
